# Bioisosteres of Carbohydrate Functional Groups in Glycomimetic Design

**DOI:** 10.3390/biomimetics4030053

**Published:** 2019-07-28

**Authors:** Rachel Hevey

**Affiliations:** Molecular Pharmacy, Department Pharmaceutical Sciences, University of Basel, Klingelbergstr. 50, 4056 Basel, Switzerland; rachel.hevey@unibas.ch

**Keywords:** bioisostere, carbohydrate, drug design, glycomimetic, lectin

## Abstract

The aberrant presentation of carbohydrates has been linked to a number of diseases, such as cancer metastasis and immune dysregulation. These altered glycan structures represent a target for novel therapies by modulating their associated interactions with neighboring cells and molecules. Although these interactions are highly specific, native carbohydrates are characterized by very low affinities and inherently poor pharmacokinetic properties. Glycomimetic compounds, which mimic the structure and function of native glycans, have been successful in producing molecules with improved pharmacokinetic (PK) and pharmacodynamic (PD) features. Several strategies have been developed for glycomimetic design such as ligand pre-organization or reducing polar surface area. A related approach to developing glycomimetics relies on the bioisosteric replacement of carbohydrate functional groups. These changes can offer improvements to both binding affinity (e.g., reduced desolvation costs, enhanced metal chelation) and pharmacokinetic parameters (e.g., improved oral bioavailability). Several examples of bioisosteric modifications to carbohydrates have been reported; this review aims to consolidate them and presents different possibilities for enhancing core interactions in glycomimetics.

## 1. Introduction

The interaction of carbohydrates with proteins and cells is well established to play important roles in biology, from the maintenance of healthy tissue to the progression of diseases. The majority of cell types have a thick layer of carbohydrates at their surface which is approximately 10–100 Å thick, referred to as the glycocalyx [1,2]. This layer acts as a primary point of contact for neighboring cells and biomolecules. These interactions are critical for communicating whether a cell is ‘self’ or ‘foreign’, and play an important role in immune activation and regulation. The composition of carbohydrates often differs between healthy and diseased cells as a result of changes in the expression levels of glycosidases and glycosyltransferases which generates unique carbohydrate patterns such as truncated or hypersialylated structures [3,4,5].

Carbohydrate-binding proteins can be classified into three major groups: (i) enzymes; (ii) glycosaminoglycan (GAG)-binding proteins; and (iii) lectins. Carbohydrate-binding enzymes include glycosidases and glycosyltransferases, which play important roles in post-translational glycosylation processes. GAGs are a constituent of proteoglycans and can interact with a number of GAG-binding proteins, most notably the interaction of heparin with antithrombin which contributes to regulation of the coagulation pathway [6]. Lectins are abundant in all species and play integral roles in infection and disease, and have therefore become desirable therapeutic targets. Although highly specific for their carbohydrate targets, they are also characterized by low affinities to their native ligands (high micromolar to millimolar *K_d_* values), except for a handful of exceptions [7,8,9].

The low affinity of lectins arises from their inherent structural properties. Ligands engage the protein through only weak interactions, such as H-bonding, metal chelation, salt bridges, and weak hydrophobic interactions. Combined, these are typically insufficient in strength to compensate for the high enthalpic desolvation penalties associated with such polar substrates and shallow, solvent-exposed binding sites. High affinity ligand binding often relies heavily on hydrophobic interactions, which are clearly lacking in polyhydroxylated, polar carbohydrate substrates. Carbohydrate–lectin interactions instead rely primarily on H-bonding, where native ligands can have difficulty competing with H-bonding from bulk solvent. To compensate for these weak interactions with native substrates, lectins typically rely on multivalency to enhance affinity in which multiple carbohydrate-binding domains engage simultaneously with multiple ligands on complementary surfaces [10,11,12,13]. Multivalent interactions are collectively much stronger than the sum of their individual interactions, due to different multivalency effects which in cooperation afford enhanced affinities. Entropy is a major contributor to multivalency: once an initial binding event has occurred, subsequent ligand interactions suffer from a reduced entropic binding penalty. In addition, multivalent presentation can effectively increase the local concentration of ligand in the proximity of the receptor.

As mentioned previously, aberrant carbohydrate patterns have been associated with a number of diseases, including cancer and immune dysregulation. Even carbohydrate structures present on healthy cells play a role in disease as they are important for immune regulation and suppression, and invading organisms are known to mimic host structures to avoid immune activation. Surface carbohydrates can also be targeted by pathogens and their toxins to immobilize onto and/or gain entry into cells [14,15]. These characteristic carbohydrates present novel targets for therapeutic development but methods to improve their binding affinities must first be established. Native carbohydrates are also susceptible to hydrolysis in both the acidic pH of the gut or by endogenous glycosidases, and methods to slow down their metabolic degradation will improve their suitability as therapeutics. Due to their high polar surface area, native carbohydrate ligands are unable to passively permeate the intestinal enterocyte layer and therefore are not orally bioavailable compounds. Features of passively permeable compounds typically conform to the Lipinski and Veber rules, such that compounds should have a low molecular weight, reduced polar surface area, and few H-bonding partners [16,17]. In addition, potentially therapeutic carbohydrates are further hindered by poor binding kinetics (fast k_off_ rates leading to short residence times) and rapid renal clearance from the circulation.

### 1.1. General Strategies for Glycomimetic Synthesis

In efforts to overcome the inherently poor properties of carbohydrates as therapeutics, much interest has been focused on designing ‘glycomimetics’ which can imitate the structure and function of native glycans [18,19]. Glycomimetics contain structural modifications which render improvements in both affinity and selectivity. Several successful examples already exist of glycomimetics developed as potential therapeutics targeting both lectins and carbohydrate-binding enzymes, for example the neuraminidase inhibitors oseltamivir (Tamiflu) and zanamivir (Relenza) have already shown great success in treating influenza infection [20,21,22].

There are multiple approaches to glycomimetic design, and these have been covered in detail in a recent review [23]. Strategies to improve binding affinity include deoxygenation, in which the OH groups deemed non-essential for binding can be removed to reduce polar surface area, thereby reducing the desolvation costs associated with binding and potentially generating new hydrophobic interactions with the protein surface. Glycomimetics can also introduce adjoining fragments that target new interactions with the protein surface peripheral to the primary binding site, most commonly targeting hydrophobic aromatic or aliphatic residues. The addition of hydrophobic fragments is also associated with reduced polar surface area, and further enhances affinity by reducing associated desolvation penalties. Conformational pre-organization has also been incorporated into glycomimetic design as it can significantly reduce entropic penalties associated with ligand binding; it has been successfully applied in several glycomimetics [24,25,26,27] and additionally tends to reduce polar surface area since the stabilizing intramolecular interactions typically involve polar functional groups which then become shielded from bulk solvent. In carbohydrate-binding enzymes, pre-organization has also been largely successful through efforts toward transition state mimetics, such as the iminoglycosides where ring distortion has been utilized to mimic the planarity of oxocarbenium transition states [8]. Additionally, multivalency has been used to successfully mimic the multivalent presentation of native ligands, which can enhance affinity through chelation, statistical rebinding effects, or clustering of binding partners [28,29,30,31].

Apart from binding affinity, strategies in glycomimetic design have also targeted improvements in pharmacokinetic properties such as better bioavailability and longer serum half-lives. As carbohydrates are easily degraded, the hydrolysis can be minimized by replacing the *O*-glycoside atom with a more stable surrogate atom (e.g., C, S) or alternatively by adding electronegative substituents to the glycan ring which destabilizes the oxocarbenium transition state required for degradation [32,33,34]. Owing to their large polar surface area, carbohydrates are unable to passively permeate the intestinal membrane and are therefore poorly absorbed into circulation. To improve passive permeation, the pro-drug approach has been used in which polar functionalities (e.g., OH, CO_2_^−^) are temporarily masked by hydrophobic groups (e.g., alkyl or aryl esters) which are then cleaved by endogenous enzymes after uptake into circulation (e.g., Tamiflu) [20,21]. Biomimetic groups can also be used to replace functional groups that are prone to degradation or are associated with rapid clearance from the serum.

With regards to enhancing affinity through chemical modification, a vast majority of the efforts in glycomimetics have been focused on targeting peripheral regions of the binding site to generate novel interactions, for example the well-studied FimH antagonists which target a ‘tyrosine gate’ [35,36,37,38]. Although this approach has proven successful, it is worthwhile to also consider bioisosteric changes that can be made to carbohydrate functional groups (OH, NHAc, etc.) and how these can be used to improve PK/PD properties. Bioisosteric changes can potentially improve both binding affinity (e.g., reduced desolvation costs, enhanced metal chelation) and pharmacokinetic parameters (e.g., modification of groups prone to metabolic degradation, improved oral bioavailability). Examples of bioisosteric modifications are scattered throughout the literature, and this review aims to consolidate them to present a range of possibilities for enhancing core interactions in glycomimetics. A summary of the strategies outlined in this review is presented in Table 1.

### 1.2. Affinity Enhancement Versus Enzyme Activity

It is important to emphasize beforehand that using biomimetic functional groups to enhance binding affinities can have variable biological consequences, especially in enzymatic carbohydrate-binding proteins. For interactions where no chemical transformation occurs upon binding, such as those involving lectins, an enhancement in target binding affords lower *K*_d_ values and typically stronger biological responses. In glycomimetics serving as antigens in carbohydrate-based conjugate vaccines, non-native ligands can afford enhanced immunogenicity over their native counterparts, resulting in elevated antibody titers and improved vaccine constructs [32,39,40,41].

Alternatively, biomimetic functional groups have been shown to have drastically different effects on enzymes. Although binding to a target can be enhanced by biomimetic replacement, its influence on the transition state of the enzymatic process can dictate whether it behaves as a substrate or an inhibitor of the enzyme. Inhibitors can be generated by directly modifying the functional group involved in the enzymatic transformation so that the ligand binds but no longer has the functionality required for the reaction. In glycosidases and related enzymes, the addition of an electron-withdrawing substituent can have profound effects on the enzymatic rate due to its destabilization of the oxocarbenium intermediate, where the position of the electron-withdrawing substituent can influence the degree of destabilization as inductive effects quickly diminish with distance [32,33,34].

## 2. Modifications to the *O*-Glycoside Linkage

Native *O*-glycan structures are sensitive to both chemical and enzymatic hydrolysis in vivo which can limit their oral bioavailability and lifetime in circulation, and subsequently their use in therapeutics. A common strategy used in glycomimetics to improve stability has been to replace the oxygen atom of the glycosidic linkage with a less labile atom or group, such as nitrogen, carbon, sulfur, or selenium [42,43,44,45,46,47,48,49,50,51]. Although these analogues impart enhanced stability, they can also introduce conformational changes which increase the entropic barrier of binding causing losses in affinity; often a desirable balance between stability and activity must be established. These conformational changes can arise from a combination of larger atom (e.g., S, Se) or loss of the exo-anomeric effect due to replacement with a less electronegative substituent (e.g., C). This conformational flexibility can potentially be reduced through the addition of steric bulk that constrains the molecule to a particular conformation [52,53,54], or by the addition of electronegative atoms such as fluorine to *C*-glycosides to reestablish the electron-withdrawing character of the anomeric substituent [51,55,56,57,58].

Aside from the more conventional isosteres listed above, the glycosidic linkage has also been replaced by other functionalities (sulfones, sulfoxides), or the linkage position has been varied to non-natural analogues which has enhanced stability [59,60,61,62]. As this has been a very well covered area of glycomimetics with many prominent examples, it has not been covered further in this review.

## 3. Replacement of the Endocyclic O Atom

In modifying glycan acetals the endocyclic oxygen atom can also be exchanged, for example, to form iminosugars, carbasugars, or thiosugars [44,63,64,65,66,67,68,69,70]. In fact, iminosugars are found naturally and widespread among different plants and microorganisms [71,72]. Hemiaminals can be unstable and therefore the lack of a C-1 oxygen-bearing substituent has been useful for improving the structural stability of these derivatives. On account of the endocyclic N atom which becomes positively charged at physiological pH, this class of molecules can mimic the charged oxocarbenium transition state observed in glycan-processing enzymes. Deoxynojirimycin is a well-studied naturally abundant iminosugar from which several drugs have been developed, including miglitol (for type II diabetes) and miglustat (*N*-butyl-1-deoxynojirimycin; for lysosomal storage disease) [72,73].

An endocyclic-*S*-containing derivative of α-GalCer (KRN7000) was synthesized for use as an adjuvant, and was observed to have a stronger immunostimulatory effect on IFN-γ production in invariant natural killer T cells as compared to its endocyclic-*O*-containing counterpart [74]. In combination with perfluoroalkyl lipid modification, a selective modulation of the immune response could be achieved where the glycomimetic 5-thio-α-galactopyranosyl-*N*-perfluorooctanoyl phytosphingosine was shown to selectively stimulate T_H_1 cytokine production, while its perfluorotetradecanoyl derivative elicited a T_H_2 cytokine profile.

**Figure 1:**
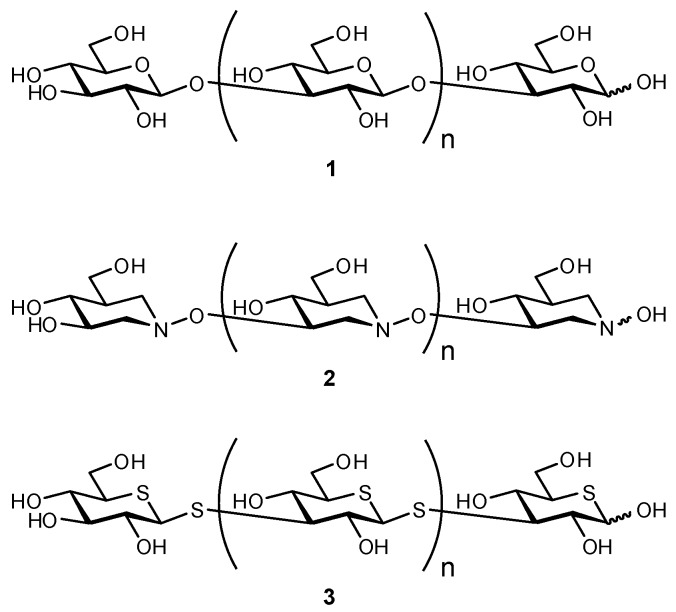
Oligosaccharide analogues to mimic β-(1→3)-glucan were synthesized as inhibitors of complement receptor 3 (CR3) and dectin-1, targeting the regulation of phagocytosis [75,76].

5-Thiopyranosyl rings were also used in the preparation of a series of di-, tri-, and tetrasaccharide β-(1→3)-glucan analogues (Figure 1) [75]. Upon evaluation of their binding to complement receptor 3 (CR3) and dectin-1 via an inhibitory assay, it was observed that the trisaccharide derivative of **3** was most optimally sized. This was a surprising observation given that the native substrate (**1**) requires much larger oligomers for effective binding. This enhancement in affinity is believed to arise from the more hydrophobic nature of S as compared to O, which enhances hydrophobic interactions with the protein. Although desirably more hydrophobic in nature, C–S bonds are also longer and therefore can result in steric mismatch of the binding partners. The related hydroxylamine analogues (**2**) were also synthesized and showed comparable affinity to the 5-thiopyranosyl derivatives [75,76]. The di-and tri-hydroxylamine mimetics were both shown to inhibit CR3 and dectin-1 binding, which was again attributed to the increase in hydrophobic nature resulting from the more polarizable hydroxylamine moiety and lack of a C-2 substituent [76,77,78], which could then interact more strongly with hydrophobic residues in the binding site (e.g., Trp221 and His223 in dectin-1 [79]).

**Figure 2:**
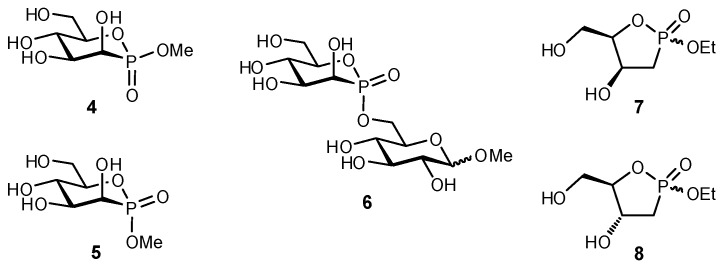
A series of phostone analogues have been evaluated as glycomimetics [80,81].

Cyclic phosphonates (‘phostones’) and phosphinates (‘phostines’) have also been used as glycomimetics, with the phosphinolactone group (O–P=O) functioning as a bioisostere of hemiacetals (O–C–OH). Phostones have been used as mimetics of both furanosides and pyranosides, where a phosphorous atom replaces the anomeric carbon (**4**–**8**; Figure 2) [80,81]. Similar to phostones, phostines can additionally impart an enhanced stability owing to their *C*-glycoside-character, and have been used to generate substrates that were antiproliferative against rat glioma tumor cells or used as antidepressants [82,83]. Phosphinolactone is a good metal chelator and H-bond acceptor; evidence supports that 6-membered rings containing phosphorus adopt stereoelectronic-dependent interactions comparable to the anomeric effect [84].

Carbasugars have also been developed as glycan mimetics, where the *O*-endocyclic atom is replaced by a C atom [69]. 5a’-Carbamaltoses were synthesized where a CH_2_ group replaced the endocyclic oxygen, with the derivatives glycosidically linked via imino, ether, or sulfide linkages [85]. The derivatives were based on the α-glucosidase inhibitor methyl acarviosin and the *N*-linked glycoside showed moderate inhibition of yeast α-glucosidase [85,86]. Cellotriose and cellotetraose derivatives were also synthesized where one of the rings had been replaced by a cyclohexene moiety, in efforts to mimic the sp^2^-hybridized transition state of cellulase hydrolysis [87]. The cyclohexene mimetics were indeed shown to effectively bind an endoglucanase (NCE5) from *Humicola insolens* and were observed to bind stronger than their native pyranose analogues.

## 4. Replacement of OH Functional Groups

Bioisosteric replacement of functional groups is commonly used in medicinal chemistry to improve the desired properties of a molecule. Biomimetic functional groups should have similar electronic and steric properties to the original group, but often different bioisosteric groups are appropriate for different situations depending on the key interactions involved (Table 2). Which bioisostere to use depends on the requirements of that particular functional group in that specific binding event with regards to, for example, steric restrictions or H-bond donors/acceptors.

Common bioisosteres of the OH group include F, SH, SeH, and NH_2_, although on alkyl scaffolds the latter becomes charged at physiological pH and therefore poorly mimics the neutral OH group. OH groups involved in metal chelation can be replaced with functionalities that act as improved metal ligands, which can be used to enhance binding affinity. Several different bioisosteric replacements have been used in the generation of glycomimetics and are discussed in the context of this review.

It is important to keep in mind that the modification of carbohydrates with functional groups that are more electron-withdrawing (e.g., OH to F, CO_2_^−^ in uronic acids), regardless of position, has been shown to increase stability of and preference for the α-anomer [88]. Although somewhat counter-intuitive, since electron-withdrawing groups reduce electron density on the endocyclic-*O*-atom and thereby would reduce the strength of the endo-anomeric effect, it is suggested that these electron-withdrawing substituents can reduce steric gauche interactions and enhance favorable Coulombic interactions between the C-1 and C–H ring substituents [88].

### 4.1. Deoxygenation

The deoxygenation of OH moieties that are non-essential for binding has shown to be a very useful method for enhancing binding affinities. Deoxygenation removes a polar functional group, which reduces polar surface area and its associated desolvation penalty, and in addition can generate new hydrophobic contacts with the protein. Studies support that at least two H-bonds to an OH group are required for a favorable binding free energy in shallow, solvent-exposed binding sites, in which case the gain in enthalpy afforded by ligand interactions becomes stronger than the enthalpic cost associated with desolvation of the polar OH group [89,90]. As deeply buried binding sites tend to be more hydrophobic, the strength of single H-bonds in these systems is enhanced. Deoxygenation at the C-6 position of pyranoses is even more favorable as it increases hydrophobicity of the molecule, and also removes a degree of freedom which lowers the entropic cost of binding.

The substitution of an OH group for H has other effects on the ligand properties. In general, deoxygenation removes an electron-withdrawing substituent and therefore increases electron density on the glycan scaffold, which can make other OH substituents more nucleophilic and potentially strengthen (or weaken) neighboring interactions such as metal coordination or H-bonding. OH groups that are not in direct contact with the protein can also be involved in pre-organization of the ligand; if this pre-organization is disturbed, then it can increase the entropic penalty of binding and therefore it may be more beneficial to retain the substituent or replace it with an alternative biomimetic group (e.g., fluorine).

Diverse methods have been used for synthesizing deoxygenated mimetics [91]. Deoxygenated derivatives have also been widely used in activity studies to gain structural information on the importance of different OH groups in receptor binding [92,93].

### 4.2. Deoxyfluorination

Fluorine is a commonly utilized bioisostere of the OH group, due to its polarity and similarity in size. The van der Waals radius of F (1.47 Å) is between that of H (1.2 Å) and O (1.57 Å) making it a suitable mimic of both [94]. Fluorine is also more hydrophobic than oxygen, and as a bioisostere of OH groups it reduces the polar surface area and binding-associated desolvation penalties and can also enhance oral bioavailability by improving passive permeation through the intestinal membrane. Although hydrophobic, it still generates a polar C–F bond that can undergo polar interactions with the protein, although typically of weaker strength. The substitution of F for OH has been widely studied, both in structural analyses of binding sites (to evaluate H-bond donor and acceptor requirements) and in the synthesis of therapeutic ligands [64,95,96,97,98].

Given the significant electronegativity of fluorine, its inductive ability can strongly destabilize oxocarbenium transition states in carbohydrate-binding enzymes and significantly slow enzymatic rates such that fluorinated glycomimetics act as excellent inhibitors of these enzymes. This was exemplified in the synthesis of 4-fluorinated derivatives of GlcNAc 1-phosphate (and related per-acetylated derivatives) which were used to reduce *N*-glycan expression on human prostate (PC-3) cancer cells and inhibit cell-growth, although the two effects appeared to arise from inhibition of two different pathways [99]. In the field of diagnostics, [^18^F]-fluorinated derivatives have shown wide applicability in positron emission tomography (PET), both in the detection of cancer as well as in newer applications such as the imaging of infections [100,101,102].

2-Deoxy-2-fluoro derivatives of two Phlorizin analogues were prepared and evaluated as inhibitors of the sodium-glucose co-transporters (SGLT1 and SGLT2) in the search for a novel therapy against type II diabetes [95]. SGLT2 is a glucose transporter located in the renal proximal tubule [103,104] which plays an important role in glucose reabsorption—by inhibiting the transporter more glucose is eliminated instead of reabsorbed, thereby decreasing glucose levels in circulation [105]. Phlorizin is a natural product isolated from apple tree bark, which inhibits both SGLT1 (causing adverse effects [106,107]) and SGLT2 and is also prone to hydrolysis, therefore improved mimetics are desirable. 2-Fluorinated analogues of SGLT2 inhibitors were evaluated for their activity and improved resistance to hydrolysis [95]; although a substrate was shown to be selective for SGLT2, it was surprisingly found to be metabolized much more quickly, presumably due to its higher lipophilicity which made it a better substrate for metabolic enzymes.

Deoxyfluorination has also been widely used in the synthesis of haptens for the preparation of carbohydrate-based conjugate vaccines. Non-natural epitopes can often be more immunogenic than native glycans, and given the similarity between the F and OH groups the antibodies elicited by non-natural epitopes are often cross-reactive with the natural targets. A small library of fluorinated trisaccharide antigens based on lipophosphoglycan from *Leishmania donovani* were synthesized in efforts to develop a vaccine against leishmaniasis [108]. Different fluorinated derivatives of β-Gal-(1→4)-[α-Man-(1→2)]-α-Man were evaluated to identify an appropriate fluorination pattern capable of improving hydrolytic stability and enhancing immunogenicity, yet still generating antibodies cross-reactive with the native glycan. A conjugate vaccine based on mucins also utilized deoxyfluorination—although the native glycopeptides were shown to have good immunogenicity, they were rapidly degraded in vivo and therefore an improvement in stability was necessary [109]. Glycomimetic derivatives with fluorine at C-6, C-2′, or C-6′ of Tn, TF, and 6-sialyl-TF antigens were synthesized and incorporated into MUC1 sequences containing a functional handle for subsequent incorporation into conjugate vaccines. Fluorinated haptens have been used in a number of other examples to improve both immunogenicity and hydrolytic stability [110,111,112].

Interestingly, one study on fluorinated GM4 derivatives reported a significant change in biological effects between fluorinated and non-fluorinated analogues [113]. GM4 is a sialosylgalactosylceramide ganglioside which is a major constituent of myelin sheath. Biological studies indicated that the natural substrate had no effect on cell viability in myelin basic protein (MBP^+^) oligodendrocytes, while the fluorinated derivative surprisingly upregulated oligodendrocyte differentiation and is being further evaluated as a therapeutic agent to inhibit demyelination, potentially offering a therapy for re-myelination of nerves in individuals affected with multiple sclerosis.

### 4.3. Methyl Etherification

Monomethyl ethers have also been used as mimics of the OH group, and widely used in activity studies to assess the binding requirements for ligand interactions. For example, each of the different monomethyl ethers of β-lactoside were synthesized and evaluated as ligands for both ricin and agglutinin, two galactoside-specific lectins isolated from *Ricinus communis* seeds [114]. In another study, a library of double-modified xyloside analogues were synthesized [115]. The library was comprised of the different combinations of monomethyl etherified, epimerized, deoxygenated, and deoxyfluorinated substitutions at the C-2 and C-4 positions with an anomeric β-linked naphthyl group, and were evaluated for their galactosyltransferase activity.

**Figure 3:**
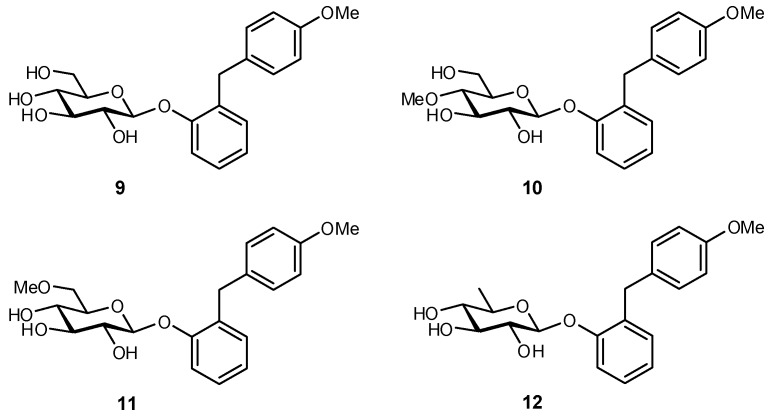
Sergliflozin-A (**9**) is an inhibitor of sodium glucose co-transporter 2 (SGLT2) but is ineffective as a therapeutic against diabetes due to its poor metabolic stability. A library of Sergliflozin-A derivatives identified the 4-*O*-methylated derivative **10** as having similar potency to Sergliflozin-A but enhanced pharmacokinetic stability [118].

As mentioned previously, the sodium glucose co-transporter 2 (SGLT2) is a novel target for lowering glucose levels in diabetic patients since inhibitors have been shown to potentially lower the renal reabsorption of glucose and reduce the risk of hyperglycemia [116,117]. In a recent study, a library of Sergliflozin-A (**9**) derivatives were prepared with various substitutions on the glucose fragment (methyl etherification, deoxygenation, deoxyfluorination) (Figure 3) [118]. The 4-OMe derivative (**10**) displayed similar potency to Sergliflozin-A (73% of the inhibition) in a rat urinary glucose excretion experiment, and with significantly improved pharmacokinetic stability. The 6-deoxy analogue **12** was also shown to be a mild inhibitor, while derivatives with substitutions at C-2 and C-3 were inactive.

Although potency was retained for the above Sergliflozin-A derivative, methyl etherification of carbohydrate hydroxyl groups is typically not the best choice of bioisosteric replacement. The CH_3_ group is much larger than an H and can introduce unfavorable steric clashes, and in addition the methyl ether group is unable to act as an H-bond donor in ligand–protein interactions. One study which demonstrates this incompatibility was aiming to synthesize inhibitors of polypeptide-α-GalNAc-transferase, an enzyme which catalyzes the first step of *O*-glycan biosynthesis [119]. Derivatives of UDP-GalNAc which were methylated at either the 3-, 4-, or 6-position were not substrates for the enzyme and displayed only very weak activity as competitive inhibitors.

### 4.4. SH and SeH Substitution

Thiosugars have been reported as naturally occurring products, although few examples of free thiol-containing structures exist such as the antibiotics rhodonocardin A and B isolated from a *Nocardia* species [120]. Both S and Se atoms are commonly used bioisosteres of O. Although sharing comparable electronic properties, S and Se are both larger atoms which results in longer C–S/Se bonds and different bond angles. These changes can have a negative impact on steric matching with the native substrates. Although atom size varies, the larger size and polarizability of S and Se atoms results in an enhanced lipophilicity which can improve hydrophobic interactions with the protein and reduce desolvation requirements of ligand binding. These mimetics are also worse H-bond donors as compared to OH groups, but non-covalent π-interactions are typically stronger with S than with O [121,122].

Thiol or selenol replacement of carbohydrate OH functional groups is not entirely common, likely given the difficulty in synthesis and isolation of these compounds caused by their propensity to dimerize into disulfides and diselenides. Examples of both dimers and alkylated thio-/selenoether derivatives have been presented in the literature and studied for various biological applications, such as their adjuvant/immunostimulatory abilities, carcinostatic activities, and antioxidant properties [123,124,125].

**Figure 4:**
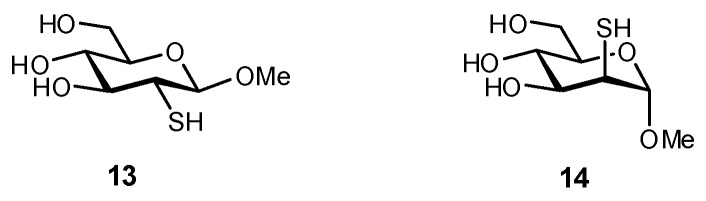
2-Thiocarbohydrates were evaluated as ligands of the plant lectin ConA [126].

Several methods have been established to synthesize thiosugars to obtain glycomimetics such as **13** and **14** (Figure 4) [126]. As it was previously reported that *S*-linked pseudodisaccharides bound better to the plant lectin concanavalin A (ConA) than their respective *O*-derivatives [60], isothermal titration calorimetry (ITC) and saturation transfer difference (STD) NMR experiments were used to evaluate the binding of these 2-thioglycosides to ConA. Surprisingly, the 2-deoxy-2-thio-mannoside derivative **14** was observed to bind ConA much better than Man itself [126,127], although STD NMR experiments suggested that this enhancement in affinity is actually the result of a slightly altered binding mode.

**Figure 5:**
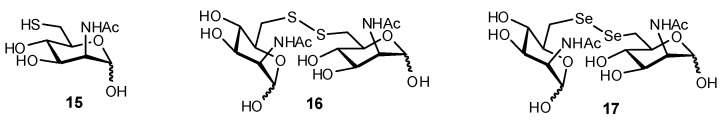
6-*S*- and 6-*Se*-derivatives of ManNAc were synthesized and evaluated as inhibitors of human *N*-acetylmannosamine kinase (MNK). The high oxidation potential of Se prevented isolation of its monomer [128].

In another study, 6-thio and 6-seleno derivatives of ManNAc were synthesized and evaluated as inhibitors of human *N*-acetylmannosamine kinase (MNK) (Figure 5) [128]. It was hypothesized that these analogues would still bind to the enzyme, yet could not be phosphorylated and therefore would act as inhibitors of the native substrate. 6-*O*-Ac-ManNAc was previously reported as an inhibitor of the enzyme [122,129,130] but its ester is easily hydrolyzed in vivo making it a poor therapeutic option. In synthesizing the 6-substituted ligands, the high oxidation potential of selenols prevented isolation of the monomer and as a result only diselenide dimer **17** could be evaluated [128]; for the thiol derivative, both monomer **15** and dimer **16** could be isolated and evaluated. Both the presence of Se and the dimerization itself provided enhancements in activity; the diselenide **17** inhibited MNK in the low μM range and upon treatment of Jurkat cells a reduction in surface sialylation was also observed.

### 4.5. Other Modifications

**Figure 6:**
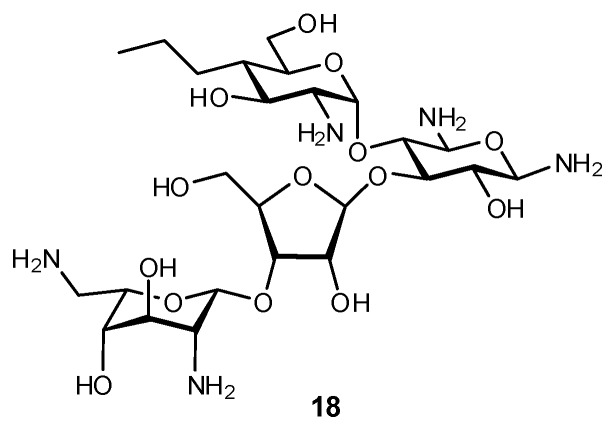
Propylamycin (**18**) is a glycomimetic that has shown excellent antibiotic efficacy against several important drug-resistant Gram-negative pathogens. The propyl substituent is believed to enhance nucleophilicity of the endocyclic O atom and thereby enhance the interaction with its electrophilic binding partner [131].

Propylamycin (**18**) is a 4′-deoxy-4′-alkyl paromomycin which is demonstrably effective against a broad selection of ESKAPE pathogens (Figure 6) [131]. Early studies had demonstrated that 4′-*O*-ethylation of paromomycin afforded enhanced ribosomal selectivity and reduced ototoxicity in antibiotic treatment [132], with the latter being one of the major negative side effects correlated with aminoglycoside therapies. Although less ototoxic, this modification was also correlated with a loss in activity. In contrast, 4′-deoxygenated paromomycin maintained antibacterial activity but had no enhancement in ribosomal selectivity. Based on these results, it was hypothesized that the alkyl derivatization with an electron-donating substituent could afford both the desired selectivity while maintaining antibacterial activity. Indeed, replacement of the 4′-OH group with a propyl chain interfered with protein synthesis in bacteria and also reduced inner ear toxicity in a guinea pig model [131]. This activity is believed to arise from the electron-donating alkyl substituent which makes the pyranose ring more electron-rich and its endocyclic O atom more nucleophilic and therefore a better H-bond acceptor, enhancing the ‘pseudo’ base pair interaction with A1408 upon binding.

## 5. Replacement of H Atoms

### 5.1. Fluorination

The small size, chemical inertness, and inherent hydrophobicity of F all contribute to it being a good bioisostere of H. Although sterically similar, its strong inductive ability can substantially influence neighboring functional groups. For example, F substitution of a Neu5Ac C-3 proton was used to inhibit sialyltransferase activity, since its inherent electron-withdrawing capability significantly increases electrophilicity of the C-2 atom and destabilizes the oxocarbenium transition state. This destabilization hinders its formation, significantly slowing the enzymatic rate and effectively reducing tumor growth in vivo [133,134]. A C-2 difluorinated derivative of α-d-glucopyranosyl-1-phosphate was also synthesized and evaluated as a substrate of thymidylyltransferase Cps2L and guanidylyltransferase GDP-ManPP [135].

**Figure 7:**
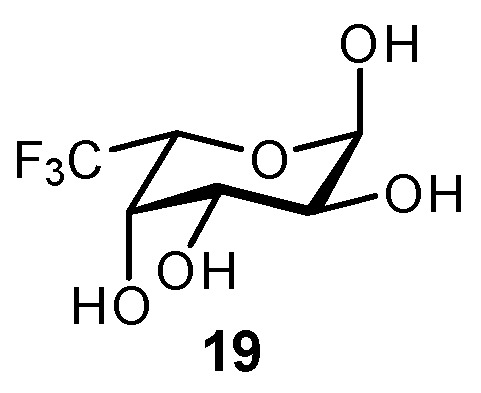
6,6,6-Trifluoro-α-fucose (**19**) was synthesized and utilized as an inhibitor of fucosylation on monoclonal antibodies [137].

The CF_3_ group has been shown to be an excellent mimic of CH_3_ in Fuc and related derivatives (**19**; Figure 7). The enhanced hydrophobicity imparted by CF_3_ reduces desolvation costs associated with binding and can enhance hydrophobic interactions with the protein surface. To date, the synthesis of CF_3_ derivatives has proven challenging and typically requires very lengthy synthetic sequences [136,137]. 6,6,6-Trifluoro-α-fucose has shown good utility as a fucosylation inhibitor during the production of therapeutic monoclonal antibodies, by binding at an allosteric site and inhibiting GDP-mannose 4,6-dehydratase [137,138]. With reduced fucosylation on the Asn297 glycan structures of IgG1 antibodies, the Fc region can bind more strongly to FcγRIIIa on effector cells, which induces enhanced antibody-dependent cellular cytotoxicity. Fucosylation inhibitors could also be useful in a number of disease therapies, including cancer [139], arthritis [140], and sickle cell disease [141].

### 5.2. Other Modifications

The replacement of H is also possible using other small, hydrophobic functional groups. For example, a small library of sialyl Lewis X derivatives were synthesized with the C-6 methyl group of Fuc modified (alkyne, fluoro, hydroxy, and hydroxymethyl) [142]. The syntheses were achieved using a recombinant l-fucokinase/GDP-fucose pyrophosphorylase and an α-(1,3)-fucosyltransferase [143]. Sialyl Lewis X has a very rigid structure, due to an important interaction involving hydrophobic stacking of the Fuc and Gal residues, where the C-5 Fuc methyl forms important van der Waals interactions with the hydrophobic face of Gal [144]. It was hypothesized that an alkynyl substitution on C-6 Fuc would enhance this pre-organization since the alkynyl group would undergo even stronger van der Waals interactions [142]. The sialyl Lewis X derivatives were observed to bind to each of the E-, L-, and P-selectins and are reportedly undergoing further study.

**Figure 8:**
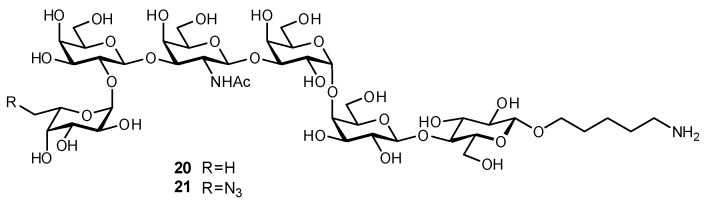
The chemoenzymatic synthesis of a 6-azido-Fuc Globo H derivative (**21**) afforded a glycomimetic which when incorporated into a conjugate vaccine elicited a strong IgG immune response in mice. The elicited antibodies were cross-reactive with the native Globo H antigen and mediated complement-dependent cell cytotoxicity against MCF-7 tumor cells [145].

The Globo H epitope has been used in clinical trials of several carbohydrate-based conjugate vaccines targeting late stage breast cancer, ovarian cancer, and prostate cancer [145]. In order to improve efficacy, it was desired to create a glycomimetic which would be more immunogenic yet still elicit antibodies cross-reactive with the native antigen present on tumor cells. Globo H derivatives were prepared with modification at either C-6 of the reducing end Glc moiety (F, N_3_, OPh, *O*-*p*-nitrophenyl, NO_2_ derivatives) or on the C-6 methyl group of Fuc (OH, N_3_, F, alkyne derivatives) [145]. Upon synthesis, the glycomimetic analogues were conjugated to a diphtheria toxoid cross-reactive material (CRM) 197 carrier protein, and then together with the adjuvant glycolipid C34 were injected into Balb/c mice. IgG antibodies elicited from several of the Glc derivatives as well as the 6-N_3_-Fuc derivative (**21**; Figure 8) were shown to be cross-reactive with Globo H and its related epitopes Gb5 and SSEAS4, as measured in a glycan array. The antibodies also recognized cell surface structures on Globo H-expressing MCF-7 tumor cells and could affect complement-dependent cytotoxic responses.

## 6. Replacement of NHAc Substituents

### 6.1. C-Derivatives

**Figure 9:**
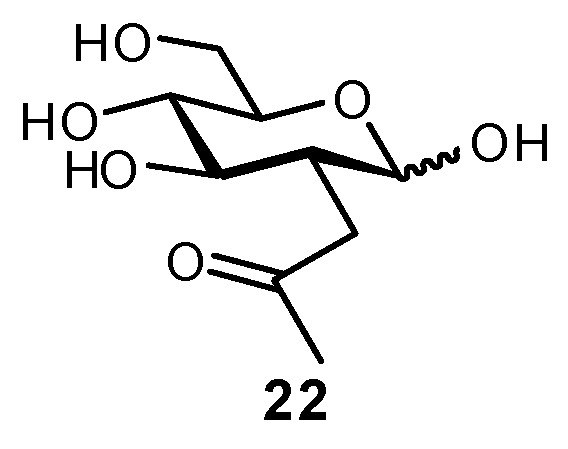
2-Deoxy-2-acetonyl derivatives were synthesized for use in glycan metabolic engineering [146].

A number of biomimetic approaches have been used to replace *N*-acetyl substituents on glycans, with most methods involving a modification at C-2. In an early example, the NHAc groups of GlcNAc and GalNAc were replaced with a ketone bioisostere in an attempt at glycan metabolic engineering of cell surfaces (**22**; Figure 9) [146,147,148]. The analogues could be fed to growing cells, which would then be incorporated onto the cell surface and could be used as a handle for conjugation to dyes, probes, etc. by reaction with aminooxy or hydrazide-bearing molecules. Upon treatment of cells, 2-*C*-modified GalNAc derivatives were taken up and detected at the surface of CHO cells, but unexpectedly were not taken up by the three human cell lines examined [147]; the GlcNAc derivative was not observed on any of the cell types.

**Figure 10:**
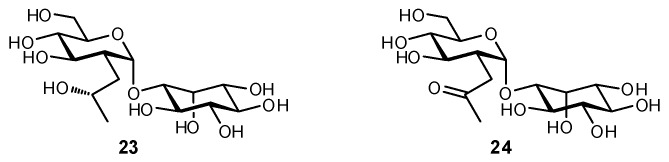
2-Deoxy-2-*C*-alkylglucosides of *myo*-inositol were synthesized as inhibitors of a *Mycobacterium smegmatis* de-*N*-acetylase [150].

Mycothiol is a 1-d-1-*O*-[2-*N*-acetamido-l-cysteinamido)-2-deoxy-α-d-glucopyranosyl]-*myo*-inositol which appears to play an important role in actinomycetes in protection from toxic agents and in preventing on oxidative intracellular environment, similar to the role of glutathione [149]. The disaccharide 1-d-1-*O*-(2-acetamido-2-deoxy-α-d-glucopyranosyl)-*myo*-inositol is an intermediate in *Mycobacteria* mycothiol production, and inhibitors of an *N*-deacetylase enzyme could potentially be used as a treatment against infection. Derivatives in which the NHAc group of the GlcNAc moiety was replaced by a C-2 substituent were synthesized and evaluated as inhibitors (Figure 10) [150]. Both 2-deoxy-2-*C*-(2′-hydroxypropyl)-d-glucoside (**23**) and 2-deoxy-2-*C*-(2′-oxopropyl)-d-glucose (**24**) derivatives successfully inhibited the production of inositol-containing metabolites, as measured by the incorporation of radiolabeled [^3^H]inositol into molecules, but unexpectedly did not affect the growth of *Mycobacterium smegmatis* in vitro.

**Figure 11:**
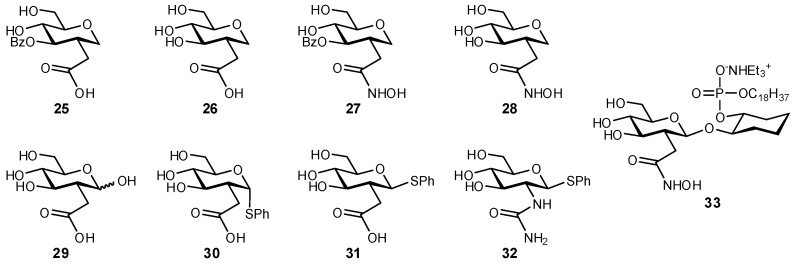
A series of 2-deoxygenated 2-*C*-modifed glucosides containing Zn^2+^-chelating groups were synthesized and evaluated as inhibitors of *Trypanosoma brucei N*-acetylglucosamine-phosphatidylinositol de-*N*-acetylase as a potential novel therapy for African sleeping sickness [151].

A number of NHAc biomimetics have also been directed at enhancing interactions with important metal ions present in the binding site, in efforts to introduce stronger chelating functionalities. *Trypanosoma brucei N*-acetylglucosamine-phosphatidylinositol (GlcNAc-PI) de-*N*-acetylase is a zinc metalloenzyme involved in the biosynthesis of glycosylphosphatidylinositol [151]. *T. brucei* is rich in GPI-anchored proteins and it is believed that disrupting their synthesis may offer a potential therapy for African sleeping sickness [151,152]. A computational homology model based on two other GlcNAc-PI de-*N*-acetylases supports a binding mode where the NHAc group is involved in coordination of a Zn^2+^ ion [153]. In efforts to install a better Zn^2+^-binding group at the C-2 position of GlcNAc, a series of analogues were prepared and evaluated for their ability to inhibit the de-*N*-acetylase (Figure 11) [151,154]. Both carboxylic acid (**25**, **29**, **31**) and hydroxamic acid (**27**, **33**) derivatives demonstrated enzyme inhibition with moderate IC_50_ values (0.1–1.5 mM) [151]. A similar approach to enhanced metal chelation was used for developing inhibitors of the de-*N*-acetylase PgaB, which is known to coordinate to the 2-NHAc and 3-OH substituents of GlcNAc [155]. Both the groups at C-3 (–OH, –SH, –NH_2_, and –N_3_) and C-2 (–NHC(=S)CH_3_), –NHS(=O)(=O)CH_3_, –CH_2_P(=O)(–OH)OMe, and –CH_2_S(=O)(=O)NH_2_) were modified and screened as inhibitors of de-*N*-acetylases which play an important role in biofilm maintenance [155]. The strongest inhibitor, 2,3-dideoxy-2-thionoacetamide-3-thio-β-d-glucoside displayed a *K*_i_ of 2.9 ± 0.6 μM.

### 6.2. Other Substitutions

As previously mentioned, glycomimetic antigens can elicit enhanced immune responses yet still elicit antibodies which recognize the native antigens. In efforts to improve immunogenicity, a number of *N*-propionyl modified haptens have been studied based on polysialic acid, sTn, and GM3 [32,40,41,145].

## 7. Replacement of Charged Substituents

Although carboxylates are present in several important glycan building blocks, such as neuraminic acids and uronic acids, there are very few examples of CO_2_^−^ bioisosteres used in glycomimetics. Based on traditional medicinal chemistry approaches, bioisosteres of the CO_2_H group include those such as CONH_2_ or SO_3_H, but these are not prominently featured in the design of glycan-based drugs. This likely results from the importance of the carboxylate interaction in effective binding. This is supported by structural evidence in several instances, for example in the mammalian sialyltransferase ST3Gal-I where it was demonstrated that conversion of the carboxylate (IC_50_: 0.047 mM) to either an amide (IC_50_: 3.3 mM) or hydroxymethylene (IC_50_: 4.2 mM) group significantly impaired ligand binding [156,157]. Instead of the bioisosteric replacement of a single functional group, carboxylate-containing fragments have been used to mimic their analogous monosaccharide building blocks [24,124,158,159,160].

**Figure 12:**
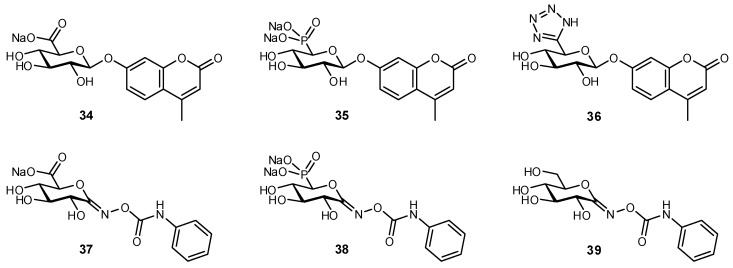
Phosphono and tetrazolyl derivatives of 4-methylumbelliferyl β-d-glucuronide were synthesized and evaluated as β-glucuronidase inhibitors. The phosphonate mimetics of the carboxylate displayed a very low level of activity, while the other mimetics were inactive [161].

In one of the few studies reported, a series of phosphono and tetrazolyl derivatives of 4-methylumbelliferyl β-d-glucuronide were synthesized and evaluated as inhibitors of β-glucuronidases (Figure 12) [161]. The phosphonate analogue **35** was observed to be slowly hydrolyzed by an *E. coli* β-glucuronidase, but was not a substrate for bovine liver β-glucuronidase. In inhibitory experiments, strong binding was observed only for the native carboxylate analogue **37** (IC_50_: 0.2 μM) and only very weak inhibition for phosphonate **38** (IC_50_: 2 mM).

Other examples of bioisosteres of carbohydrate derivatives containing charged modifications include the installation of a carboxylate group at C-1 of lipid A to mimic the native phosphoric acid moiety [162]. Furthermore, 6-bromophosphonate analogues of glucose-6-phosphate were synthesized and demonstrated to inhibit glucose-6-phosphatase [163]. Glucose-6-phosphatase is involved in the regulation of glucose metabolism and is upregulated in type II diabetes [164,165], inferring that inhibitors of the enzyme may offer a potentially novel therapeutic.

## 8. Conclusions

Although native carbohydrates have traditionally been viewed as poor therapeutic agents, advancements in the development of glycomimetics have been made which has enabled carbohydrate-protein interactions to be more actively considered as drug targets. A main strategy for affinity enhancement in glycomimetics is to reduce the ligand polar surface area, which can be achieved through the bioisosteric replacement of carbohydrate core functional groups. Both deoxygenation and deoxyfluorination of OH groups have demonstrated broad successes in enhancing affinity over native substrates, and have become a major area of focus in glycomimetic development. This benefit is even further evident at the C-6 position of pyranosides, where deoxygenation removes a rotatable bond and thereby concomitantly reduces the entropic penalty of binding. With regards to fluorination, CF_3_-containing fucose derivatives have also been very successful, yet their accessibility has been limited by the difficulty encountered in their syntheses. In efforts to enhance the enthalpic gains associated with ligand binding, only limited efforts have been reported on the bioisosteric replacement of chelating groups or improved H-bond donors/acceptors, and these approaches should be evaluated further.

## Figures and Tables

**Table 1:** A summary of the different bioisosteres of carbohydrate functional groups discussed in this review, including the rationale behind the selected substitution.

Functional Group	Bioisosteric Replacement	Rationale	Disadvantages
*O*-Glycosidic linkage	*N*-, *C*-, *S*-, *Se*-Glycosides	*O*-Glycosides can be susceptible to chemical/enzymatic hydrolysis in vivo	Enhanced flexibility (larger atom, loss of anomeric effect)
Endocyclic *O* atom	Imino-, thio-, carbasugars, phostones, phostines	Enhance stability; Reduce polar surface area; Iminosugars can mimic charged oxocarbenium transition state	Changes in pyranoside conformation; Loss of anomeric effect
OH	Deoxygenation	Reduce polar surface area; Increase hydrophobic contacts with protein	Potentially disrupts critical ligand-protein interactions; Disrupts ligand pre-organization
OH	Deoxyfluorination	Similar polarity and size; H-bond acceptor ability; Reduce polar surface area; Destabilize oxocarbenium transition state	Removes H-bond donor ability
OH	Methyl etherification	Reduce polar surface area	Removes H-bond donor ability; Potential steric incompatibilities
OH	SH/SeH substitution	Reduce polar surface area (enhanced atom polarizability); Enhance π-interactions	Larger atoms; Longer bonds/altered bond angles; Weaker H-bond donors
H	Fluorination	Similar size and hydrophobicity; Chemically inert; Destabilize oxocarbenium transition state	Alters electron-density in neighboring substituents
NHAc	*C*-, *N*-derivatives	Enhance metal chelation; Introduce novel functionalities for bioconjugation (e.g., ketone)	Potentially introduces steric incompatibilities or charged substituents
CO_2_^−^	Amide, sulfonate, phosphonate	Reduce polar surface area; Enhance charged protein interactions	Disrupts critical carboxylate–protein interactions

**Table 2:** Common bioisosteres of different functional groups.

Original Group	Potential Replacements
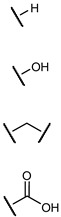	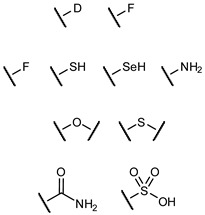

## References

[1] Nieuwdorp M., Meuwese M.C., Mooij H.L., Ince C., Broekhuizen L.N., Kastelein J.J.P., Stroes E.S.G., Vink H. (2008). Measuring endothelial glycocalyx dimensions in humans: A potential novel tool to monitor vascular vulnerability. J. Appl. Physiol..

[2] Van Teeffelen J.W., Brands J., Stroes E.S., Vink H. (2007). Endothelial glycocalyx: Sweet shield of blood vessels. Trends Cardiovasc. Med..

[3] Reily C., Stewart T.J., Renfrow M.B., Novak J. (2019). Glycosylation in health and disease. Nat. Rev. Nephrol..

[4] Ohtsubo K., Marth J.D. (2006). Glycosylation in cellular mechanisms of health and disease. Cell.

[5] Ashkani J., Naidoo K.J. (2016). Glycosyltransferase gene expression profiles classify cancer types and propose prognostic subtypes. Sci. Rep..

[6] Jin L., Abrahams J.P., Skinner R., Petitou M., Pike R.N., Carrell R.W. (1997). The anticoagulant activation of antithrombin by heparin. Proc. Natl. Acad. Sci. USA.

[7] Quiocho F.A. (1993). Probing the atomic interactions between proteins and carbohydrates. Biochem. Soc. Trans..

[8] Sears P., Wong C.-H. (1999). Carbohydrate mimetics: A new strategy for tackling the problem of carbohydrate-mediated biological recognition. Angew. Chem. Int. Ed..

[9] Schön A., Freire E. (1989). Thermodynamics of intersubunit interactions in cholera toxin upon binding to the oligosaccharide portion of its cell surface receptor, ganglioside G_M1_. Biochemistry.

[10] Mammen M., Choi S.-K., Whitesides G.M. (1998). Polyvalent interactions in biological systems: Implications for design and use of multivalent ligands and inhibitors. Angew. Chem. Int. Ed..

[11] Müller C., Despras G., Lindhorst T.K. (2016). Organizing multivalency in carbohydrate recognition. Chem. Soc. Rev..

[12] Huskens J., Prins L.J., Haag R., Ravoo B.J. (2018). Multivalency: Concepts, Research & Applications.

[13] Kiessling L.L., Young T., Gruber T.D., Mortell K.H., Fraser-Reid B.O., Tatsuta K., Thiem J. (2008). Multivalency in protein-carbohydrate recognition. Glycoscience: Chemistry and Chemical Biology.

[14] Smith D.C., Lord J.M., Roberts L.M., Johannes L. (2004). Glycosphingolipids as toxin receptors. Semin. Cell Dev. Biol..

[15] Gamblin S.J., Skehel J.J. (2010). Influenza hemagglutinin and neuraminidase membrane glycoproteins. J. Biol. Chem..

[16] Lipinski C.A., Lombardo F., Dominy B.W., Feeney P.J. (1997). Experimental and computational approaches to estimate solubility and permeability in drug discovery and development settings. Adv. Drug Deliver. Rev..

[17] Veber D.F., Johnson S.R., Cheng H.-Y., Smith B.R., Ward K.W., Kopple K.D. (2002). Molecular properties that influence the oral bioavailability of drug candidates. J. Med. Chem..

[18] Ernst B., Magnani J.L. (2009). From carbohydrate leads to glycomimetic drugs. Nat. Rev. Drug Discov..

[19] Wong C.-H. (2003). Carbohydrate-Based Drug Discovery.

[20] Kim C.U., Lew W., Williams M.A., Liu H., Zhang L., Swaminathan S., Bischofberger N., Chen M.S., Mendel D.B., Tai C.Y. (1997). Influenza neuraminidase inhibitors possessing a novel hydrophobic interaction in the enzyme active site: Design, synthesis, and structural analysis of carbocyclic sialic acid analogues with potent anti-influenza activity. J. Am. Chem. Soc..

[21] McClellan K., Perry C.M. (2001). Oseltamivir - a review of its use in influenza. Drugs.

[22] von Itzstein M., Wu W.-Y., Kok G.B., Pegg M.S., Dyason J.C., Jin B., Phan T.V., Smythe M.L., White H.F., Oliver S.W. (1993). Rational design of potent sialidase-based inhibitors of influenza virus replication. Nature.

[23] Hevey R. (2019). Strategies for the development of glycomimetic drug candidates. Pharmaceuticals.

[24] Chang J., Patton J.T., Sarkar A., Ernst B., Magnani J.L., Frenette P.S. (2010). GMI-1070, a novel pan-selectin antagonist, reverses acute vascular occlusions in sickle cell mice. Blood.

[25] Dugger S.A., Platt A., Goldstein D.B. (2018). Drug development in the era of precision medicine. Nat. Rev. Drug Discov..

[26] Kolb H.C., Ernst B. (1997). Development of tools for the design of selectin antagonists. Chem. Eur. J..

[27] Thoma G., Magnani J.L., Patton J.T., Ernst B., Jahnke W. (2001). Preorganization of the bioactive conformation of sialyl Lewis^X^ analogues correlates with their affinity to E-selectin. Angew. Chem..

[28] García-Moreno M.I., Ortega-Caballero F., Rísquez-Cuadro R., Ortiz Mellet C., García Fernandez J.M. (2017). The impact of heteromultivalency in lectin recognition and glycosidase inhibition: An integrated mechanistic study. Chem. Eur. J..

[29] Ordanini S., Varga N., Porkolab V., Thépaut M., Belvisi L., Bertaglia A., Palmioli A., Berzi A., Trabattoni D., Clerici M. (2015). Designing nanomolar antagonists of DC-SIGN-mediated HIV infection: Ligand presentation using molecular rods. Chem. Commun..

[30] Berzi A., Ordanini S., Joosten B., Trabattoni D., Cambi A., Bernardi A., Clerici M. (2016). Pseudo-mannosylated DC-SIGN ligands as immunomodulants. Sci. Rep..

[31] Compain P. (2019). Multivalent effect in glycosidase inhibition: The end of the beginning. Chem. Rec..

[32] Hevey R., Ling C.-C. (2012). Recent advances in developing synthetic carbohydrate-based vaccines for cancer immunotherapies. Future Med. Chem..

[33] Biffinger J.C., Kim H.W., DiMagno S.G. (2004). The polar hydrophobicity of fluorinated compounds. ChemBioChem.

[34] Withers S.G., MacLennan D.J., Street I.P. (1986). The synthesis and hydrolysis of a series of deoxyfluoro-D-glucopyranosyl phosphates. Carbohydr. Res..

[35] Han Z., Pinkner J.S., Ford B., Obermann R., Nolan W., Wildman S.A., Hobbs D., Ellenberger T., Cusumano C.K., Hultgren S.J. (2010). Structure-based drug design and optimization of mannose bacterial FimH antagonists. J. Med. Chem..

[36] Bouckaert J., Berglund J., Schembri M., De Genst E., Cools L., Wuhrer M., Hung C.-S., Pinkner J., Slättegård R., Zavialov A. (2005). Receptor binding studies disclose a novel class of high-affinity inhibitors of the *Escherichia coli* FimH adhesin. Mol. Microbiol..

[37] Sperling O., Fuchs A., Lindhorst T.K. (2006). Evaluation of the carbohydrate recognition domain of the bacterial adhesin FimH: Design, synthesis and binding properties of mannoside ligands. Org. Biomol. Chem..

[38] Klein T., Abgottspon D., Wittwer M., Rabbani S., Herold J., Jiang X., Kleeb S., Lüthi C., Scharenberg M., Bezençon J. (2010). FimH antagonists for the oral treatment of urinary tract infections: From design and synthesis to in vitro and in vivo evaluation. J. Med. Chem..

[39] Hevey R., Ling C.-C., Narain R. (2013). Conjugation strategies used for the preparation of carbohydrate-conjugate vaccines. Chemistry of Bioconjugates: Synthesis, Characterization, and Biomedical Applications.

[40] Krug L.M., Ragupathi G., Ng K.K., Hood C., Jennings H.J., Guo Z., Kris M.G., Miller V., Pizzo B., Tyson L. (2004). Vaccination of small cell lung cancer patients with polysialic acid or *N*-propionylated polysialic acid conjugated to keyhole limpet hemocyanin. Clin. Cancer Res..

[41] Sahabuddin S., Chang T.-C., Lin C.-C., Jan F.-D., Hsiao H.-Y., Huang K.-T., Chen J.-H., Horng J.-C., Ho J.A., Lin C.-C. (2010). Synthesis of *N*-modified sTn analogs and evaluation of their immunogenicities by microarray-based immunoassay. Tetrahedron.

[42] Yang Y., Yu B. (2017). Recent advances in the chemical synthesis of *C*-glycosides. Chem. Rev..

[43] Ati J., Lafite P., Daniellou R. (2017). Enzymatic synthesis of glycosides: From natural *O*- and *N*-glycosides to rare *C*- and *S*-glycosides. Beilstein J. Org. Chem..

[44] Horne G., Wilson F.X., Lawton G., Witty D.R. (2011). Therapeutic applications of iminosugars: Current perspectives and future opportunities. Progress in Medicinal Chemistry.

[45] Sánchez-Fernández E.M., Rísquez-Cuadro R., Ortiz Mellet C., García Fernández J.M., Nieto P.M., Angulo J. (2012). sp^2^-Iminosugar *O*-, *S*-, and *N*-glycosides as conformational mimics of α-linked disaccharides; Implications for glycosidase inhibition. Chem. Eur. J..

[46] Dondoni A., Catozzi N., Marra A. (2005). Concise and practical synthesis of *C*-glycosyl ketones from sugar benzothiazoles and their transformation into chiral tertiary alcohols. J. Org. Chem..

[47] Mydock-McGrane L., Cusumano Z., Han Z., Binkley J., Kostakioti M., Hannan T., Pinkner J.S., Klein R., Kalas V., Crowley J. (2016). Antivirulence *C*-mannosides as antibiotic-sparing, oral therapeutics for urinary tract infections. J. Med. Chem..

[48] Chalopin T., Alvarez Dorta D., Sivignon A., Caudan M., Dumych T.I., Bilyy R.O., Deniaud D., Barnich N., Bouckaert J., Gouin S.G. (2016). Second generation of thiazolylmannosides, FimH antagonists for *E. coli*-induced Crohn’s disease. Org. Biomol. Chem..

[49] Illyés T.-Z., Balla S., Bényei A., Kumar A.A., Timári I., Kövér K.E., Szilágyi L. (2016). Exploring the syntheses of novel glycomimetics. Carbohydrate derivatives with *Se*-*S*- or *Se*-*Se*- glycosidic linkages. ChemistrySelect.

[50] Zhu F., O’Neill S., Rodriguez J., Walczak M.A. (2018). Stereoretentive reactions at the anomeric position: Synthesis of selenoglycosides. Angew. Chem. Int. Ed..

[51] Hirai G., Watanabe T., Yamaguchi K., Miyagi T., Sodeoka M. (2007). Stereocontrolled and convergent entry to *CF*_2_-sialosides: Synthesis of *CF*_2_-linked ganglioside GM4. J. Am. Chem. Soc..

[52] Asensio J.L., Espinosa J.F., Dietrich H., Cañada F.J., Schmidt R.R., Martín-Lomas M., André S., Gabius H.-J., Jiménez-Barbero J. (1999). Bovine heart galectin-1 selects a unique (syn) conformation of C-lactose, a flexible lactose analogue. J. Am. Chem. Soc..

[53] Asensio J.L., Cañada F.J., Cheng X., Khan N., Mootoo D.R., Jiménez-Barbero J. (2000). Conformational differences between O- and C-glycosides: The α-*O*-Man-(1➝1)-β-Gal/α-*C*-Man-(1➝1)-β-Gal case - a decisive demonstration of the importance of the *exo*-anomeric effect on the conformation of glycosides. Chem. Eur. J..

[54] Espinosa J.-F., Bruix M., Jarreton O., Skrydstrup T., Beau J.-M., Jiménez-Barbero J. (1999). Conformational differences between *C*- and *O*-glycosides: The α-*C*-mannobiose/α-*O*-mannobiose case. Chem. Eur. J..

[55] O’’Hagan D., Rzepa H.S. (1997). Some influences of fluorine in bioorganic chemistry. Chem. Commun..

[56] Berber H., Brigaud T., Lefebvre O., Plantier-Royon R., Portella C. (2001). Reactions of difluoroenoxysilanes with glycosyl donors: Synthesis of difluoro-C-glycosides and difluoro-C-disaccharides. Chem. Eur. J..

[57] Moreno B., Quehen C., Rose-Hélène M., Leclerc E., Quirion J.-C. (2007). Addition of difluoromethyl radicals to glycals: A new route to alpha-CF_2_-D-glycosides. Org. Lett..

[58] Poulain F., Serre A.-L., Lalot J., Leclerc E., Quirion J.-C. (2008). Synthesis of α-CF_2_-mannosides and their conversion to fluorinated pseudoglycopeptides. J. Org. Chem..

[59] Mukherjee C., Ghosh S., Nandi P., Sen P.C., Misra A.K. (2010). Efficient synthesis of (6-deoxy-glycopyranosid-6-yl) sulfone derivatives and their effect on Ca^2+^-ATPase. Eur. J. Med. Chem..

[60] Cumpstey I., Ramstadius C., Akhtar T., Goldstein I.J., Winter H.C. (2010). Non-glycosidically linked pseudodisaccharides: Thioethers, sulfoxides, sulfones, ethers, selenoethers, and their binding to lectins. Eur. J. Org. Chem..

[61] Barbaud C., Bols M., Lundt I. (1995). Synthesis of the first pseudosugar-C-disaccharide. A potential antigen for eliciting glycoside-bond forming antibodies with catalytic groups. Tetrahedron.

[62] Céspedes Dávila M.F., Schneider J.P., Godard A., Hazelard D., Compain P. (2018). One-pot, highly stereoselective synthesis of dithioacetal-α,α-diglycosides. Molecules.

[63] Borges de Melo E., da Silveira Gomes A., Carvalho I. (2006). α- and β-glucosidase inhibitors: Chemical structure and biological activity. Tetrahedron.

[64] Drueckhammer D.G., Wong C.-H. (1985). Chemoenzymatic syntheses of fluoro sugar phosphates and analogues. J. Org. Chem..

[65] Mehta S., Andrews J.S., Johnston B.D., Pinto B.M. (1994). Novel heteroanalogues of methyl maltoside containing sulfur and selenium as potential glycosidase inhibitors. J. Am. Chem. Soc..

[66] Mehta S., Andrews J.S., Johnston B.D., Svensson B., Pinto B.M. (1995). Synthesis and enzyme inhibitory activity of novel glycosidase inhibitors containing sulfur and selenium. J. Am. Chem. Soc..

[67] Johnston B.D., Pinto B.M. (1998). Synthesis of heteroanalogues of disaccharides as potential inhibitors of the processing mannosidase Class I enzymes. Carbohydr. Res..

[68] Compain P., Martin O.R. (2007). Iminosugars: From Synthesis to Therapeutic Applications.

[69] Arjona O., Gómez A.M., López J.C., Plumet J. (2007). Synthesis and conformational and biological aspects of carbasugars. Chem. Rev..

[70] Witczak Z.J., Culhane J.M. (2005). Thiosugars: New perspectives regarding availability and potential biochemical and medicinal applications. Appl. Microbiol. Biotechnol..

[71] Paulsen H. (1966). Carbohydrates containing nitrogen or sulfur in the “hemiacetal” ring. Angew. Chem. Int. Ed..

[72] Gu X., Gupta V., Yang Y., Zhu J.-Y., Carlson E.J., Kingsley C., Tash J.S., Schönbrunn E., Hawkinson J., Georg G.I. (2017). Structure-activity studies of *N*-butyl-1-deoxynojirimycin (*N*B-DNJ) analogues: Discovery of potent and selective aminocyclopentitol inhibitors of GBA1 and GBA2. ChemMedChem.

[73] Inouye S., Tsuruoka T., Ito T., Niida T. (1968). Structure and synthesis of nojirimycin. Tetrahedron.

[74] He P., Zhao C., Lu J., Zhang Y., Fang M., Du Y. (2019). Synthesis of 5-thio-α-GalCer analogues with fluorinated acyl chain on lipid residue and their biological evaluation. ACS Med. Chem. Lett..

[75] Liao X., Větvička V., Crich D. (2018). Synthesis and evaluation of 1,5-dithia-D-laminaribiose, triose, and tetraose as truncated β-(1→3)-glucan mimetics. J. Org. Chem..

[76] Ferry A., Malik G., Guinchard X., Vĕtvička V., Crich D. (2014). Synthesis and evaluation of di- and trimeric hydroxylamine-based β-(1→3)-glucan mimetics. J. Am. Chem. Soc..

[77] Santana A.G., Jiménez-Moreno E., Gómez A.M., Corzana F., González C., Jiménez-Oses G., Jiménez-Barbero J., Asensio J.L. (2013). A dynamic combinatorial approach for the analysis of weak carbohydrate/aromatic complexes: Dissecting facial selectivity in CH/π stacking interactions. J. Am. Chem. Soc..

[78] Asensio J.L., Ardá A., Cañada F.J., Jiménez-Barbero J. (2013). Carbohydrate-aromatic interations. Acc. Chem. Res..

[79] Sylla B., Guégan J.-P., Wieruszeski J.-M., Nugier-Chauvin C., Legentil L., Daniellou R., Ferrières V. (2011). Probing β-(1→3)-D-glucans interactions with recombinant human receptors using high-resolution NMR studies. Carbohydr. Res..

[80] Dayde B., Pierra C., Gosselin G., Surleraux D., Ilagouma A.T., Laborde C., Volle J.-N., Virieux D., Pirat J.-L. (2014). Synthesis of unnatural phosphonosugar analogues. Eur. J. Org. Chem..

[81] Ferry A., Guinchard X., Retailleau P., Crich D. (2012). Synthesis, characterization, and coupling reactions of six-membered cyclic P-chiral ammonium phosphonite-boranes; Reactive *H*-phosphinate equivalents for the stereoselective synthesis of glycomimetics. J. Am. Chem. Soc..

[82] Clarion L., Jacquard C., Sainte-Catherine O., Loiseau S., Filippini D., Hirlemann M.-H., Volle J.-N., Virieux D., Lecouvey M., Pirat J.-L. (2012). Oxaphosphinanes: New therapeutic perspectives for glioblastoma. J. Med. Chem..

[83] Volle J.-N., Filippini D., Krawczy B., Kaloyanov N., Van der Lee A., Maurice T., Pirat J.-L., Virieux D. (2010). Drug discovery: Phosphinolactone, in vivo bioisostere of the lactol group. Org. Biomol. Chem..

[84] Hernández J., Ramos R., Sastre N., Meza R., Hommer H., Salas M., Gordillo B. (2004). Conformational analysis of six-membered ring dioxaphosphinanes. Part 1: Anancomeric thiophosphates. Tetrahedron.

[85] Tsunoda H., Sasaki S., Furuya T., Ogawa S. (1996). Synthesis of methyl 5a’-carbamaltoses linked by imino, ether and sulfide bridges and unsaturated derivatives thereof. Liebigs Ann..

[86] Junge B., Heiker F.-R., Kurz J., Muller L., Schmidt D.D., Wunsche C. (1984). Untersuchungen zur Struktur des α-D-glucosidaseinhibitors Acarbose. Carbohydr. Res..

[87] Noguchi S., Takemoto S., Kidokoro S., Yamamoto K., Hashimoto M. (2011). Syntheses of cellotriose and cellotetraose analogues as transition state mimics for mechanistic studies of cellulases. Bioorg. Med. Chem..

[88] Kerins L., Byrne S., Gabba A., Murphy P.V. (2018). Anomer preferences for glucuronic and galacturonic acid and derivatives and influence of electron-withdrawing substituents. J. Org. Chem..

[89] Sager C.P., Eriş D., Smieško M., Hevey R., Ernst B. (2017). What contributes to an effective mannose recognition domain?. Beilstein J. Org. Chem..

[90] Cabani S., Gianni P., Mollica V., Lepori L. (1981). Group contributions to the thermodynamic properties of non-ionic organic solutes in dilute aqueous solution. J. Solution Chem..

[91] Ge J.-T., Li Y.-Y., Tian J., Liao R.-Z., Dong H. (2017). Synthesis of deoxyglycosides by desulfurization under UV light. J. Org. Chem..

[92] Danac R., Ball L., Gurr S.J., Fairbanks A.J. (2008). Synthesis of UDP-glucose derivatives modified at the 3-OH as potential chain terminators of β-glucan biosynthesis. Carbohydr. Res..

[93] Wands A.M., Cervin J., Huang H., Zhang Y., Youn G., Brautigam C.A., Dzebo M.M., Björklund P., Wallenius V., Bright D.K. (2018). Fucosylated molecules competitively interfere with cholera toxin binding to host cells. ACS Infect. Dis..

[94] Bondi A. (1964). van der Waals volumes and radii. J. Phys. Chem..

[95] Sadurní A., Kehr G., Ahlqvist M., Wernevik J., Sjögren H.P., Kankkonen C., Knerr L., Gilmour R. (2018). Fluorine-directed glycosylation enables the stereocontrolled synthesis of selective SGLT2 inhibitors for type II diabetes. Chem. Eur. J..

[96] Denavit V., Lainé D., Bouzriba C., Shanina E., Gillon É., Fortin S., Rademacher C., Imberty A., Giguère D. (2019). Stereoselective synthesis of fluroinated galactopyranosides as potential molecular probes for galactophilic proteins: Assessment of monofluorogalactoside-LecA interactions. Chem. Eur. J..

[97] Karban J., Horník Š., Šťastná L.Č., Sýkora J. (2014). A convenient route to peracetylated 3-deoxy-3-fluoro analogues of D-glucosamine and D-galactosamine from a Černý epoxide. Synlett.

[98] Kasuya M.C., Ito A., Hatanaka K. (2007). Simple and convenient synthesis of a fluorinated GM4 analogue. J. Fluor. Chem..

[99] Nishimura S.-I., Hato M., Hyugaji S., Feng F., Amano M. (2012). Glycomics for drug discovery: Metabolic perturbation in androgen-independent prostate cancer cells induced by unnatural hexosamine mimics. Angew. Chem. Int. Ed..

[100] Sprinz C., Zanon M., Altmayer S., Watte G., Irion K., Marchiori E., Hochhegger B. (2018). Effects of blood glucose level on 18F fluorodeoxyglucose (18F-FDG) uptake for PET/CT in normal organs: An analysis on 5623 patients. Sci. Rep..

[101] Maschauer S., Haubner R., Kuwert T., Prante O. (2014). ^18^F-Glyco-RGD peptides for PET imaging of integrin expression: Efficient radiosynthesis by click chemistry and modulation of biodistribution by glycosylation. Mol. Pharm..

[102] Namavari M., Gowrishankar G., Srinivasan A., Gambhir S.S., Haywood T., Beinat C. (2017). A novel synthesis of 6′′-[^18^F]-fluoromaltotriose as a PET tracer for imaging bacterial infection. J. Label. Compd. Radiopharm..

[103] Bouchie A. (2013). SGLT2 inhibitors enter crowded diabetes space. Nat. Biotechnol..

[104] DeFronzo R.A., Norton L., Abdul-Ghani M. (2017). Renal, metabolic and cardiovascular considerations of SGLT2 inhibition. Nat. Rev. Nephrol..

[105] Kanai Y., Lee W.-S., You G., Brown D., Hediger M.A. (1994). The human kidney low affinity Na^+^/glucose cotransporter SGLT2. J. Clin. Investig..

[106] Kasahara M., Maeda M., Hayashi S., Mori Y., Abe T. (2001). A missense mutation in the Na^+^/glucose cotransporter gene *SGLT1* in a patient with congenital glucose-galactose malabsorption: Normal trafficking but inactivation of the mutant protein. Biochim. Biophys. Acta.

[107] Turk E., Zabel B., Mundlos S., Dyer J., Wright E.M. (1991). Glucose/galactose malabsorption caused by a defect in the Na^+^/glucose cotransporter. Nature.

[108] Baumann A., Marchner S., Daum M., Hoffmann-Röder A. (2018). Synthesis of fluorinated *Leishmania* cap trisaccharides for diagnostic tool and vaccine development. Eur. J. Org. Chem..

[109] Wagner S., Mersch C., Hoffmann-Röder A. (2010). Fluorinated glycosyl amino acids for mucin-like glycopeptide antigen analogues. Chem. Eur. J..

[110] Muldard L.A., Kováč P., Glaudemans C.P.J. (1994). Synthesis of specifically monofluorinated ligands related to the O-polysaccharide of *Shigella dysenteriae* type 1. Carbohydr. Res..

[111] Oberbillig T., Mersch C., Wagner S., Hoffmann-Röder A. (2012). Antibody recognition of fluorinated MUC1 glycopeptide antigens. Chem. Commun..

[112] Yang F., Zheng X.-J., Huo C.-X., Wang Y., Zhang Y., Ye X.-S. (2011). Enhancement of the immunogenicity of synthetic carbohydrate vaccines by chemical modifications of STn antigen. ACS Chem. Biol..

[113] Kieser T.J., Santschi N., Nowack L., Kehr G., Kuhlmann T., Albrecht S., Gilmour R. (2018). Single site fluorination of the GM_4_ ganglioside epitope upregulates oligodendrocyte differentiation. ACS Chem. Neurosci..

[114] Fernández P., Jiménez-Barbero J., Martín-Lomas M. (1994). Syntheses of all the possible monomethyl ethers and several deoxyhalo analogues of methyl β-lactoside as ligands for the *Ricinus communis* lectins. Carbohydr. Res..

[115] Willén D., Bengtsson D., Clementson S., Tykesson E., Manner S., Ellervik U. (2018). Synthesis of double-modified xyloside analogues for probing the β4GalT7 active site. J. Org. Chem..

[116] Idris I., Donnelly R. (2009). Sodium-glucose co-transporter-2 inhibitors: An emerging new class of oral antidiabetic drug. Diabetes Obes. Metab..

[117] Verspohl E.J. (2012). Novel pharmacological approaches to the treatment of type 2 diabetes. Pharmacol. Rev..

[118] Cao X., Zhang W., Yan X., Huang Z., Zhang Z., Wang P., Shen J. (2016). Modification on the *O*-glucoside of Sergliflozin-A: A new strategy for SGLT2 inhibitor design. Bioorg. Med. Chem. Lett..

[119] Busca P., Piller V., Piller F., Martin O.R. (2003). Synthesis and biological evaluation of new UDP-GalNAc analogues for the study of polypeptide-α-GalNAc-transferases. Bioorg. Med. Chem. Lett..

[120] Lin C.-I., McCarty R.M., Liu H. (2013). The biosynthesis of nitrogen-, sulfur-, and high-carbon chain-containing sugars. Chem. Soc. Rev..

[121] Berkov-Zrihen Y., Herzog I.M., Feldman M., Sonn-Segev A., Roichman Y., Fridman M. (2013). Di-alkylated paromomycin derivatives: Targeting the membranes of Gram positive pathogens that cause skin infections. Bioorg. Med. Chem..

[122] Martinez J., Nguyen L.D., Hinderlich S., Zimmer R., Tauberger E., Reutter W., Saenger W., Fan H., Moniot S. (2012). Crystal structures of *N*-acetylmannosamine kinase provide insights into enzyme activity and inhibition. J. Biol. Chem..

[123] Chen G.C., Banks C.H., Irgolic K.J., Zingaro R.A. (1980). Syntheses of 1- and 6-*S*- and 1- and 6-*Se*-derivatives of 2-amino-2-deoxy-α/β-D-glucopyranose. J. Chem. Soc. Perkin 1.

[124] Liakatos A., Kiefel M.J., Fleming F., Coulson B., von Itzstein M. (2006). The synthesis and biological evaluation of lactose-based sialylmimetics as inhibitors of rotaviral infection. Bioorg. Med. Chem..

[125] Vargas J.P., Pinto L.M., Savegnago L., Lüdtke D.S. (2015). Synthesis of alkylseleno-carbohydrates and evaluation of their antioxidant properties. J. Braz. Chem. Soc..

[126] Pavashe P., Elamparuthi E., Hettrich C., Möller H.M., Linker T. (2016). Synthesis of 2-thiocarbohydrates and their binding to concanavalin A. J. Org. Chem..

[127] Wu B., Ge J., Ren B., Pei Z., Dong H. (2015). Synthesis and binding affinity analysis of positional thiol analogs of mannopyranose for the elucidation of sulfur in different position. Tetrahedron.

[128] Nieto-Garcia O., Wratil P.R., Nguyen L.D., Böhrsch V., Hinderlich S., Reutter W., Hackenberger C.P.R. (2016). Inhibition of the key enzyme of sialic acid biosynthesis by C6-Se modified *N*-acetylmannosamine analogs. Chem. Sci..

[129] Blume A., Chen H., Reutter W., Schmidt R.R., Hinderlich S. (2002). 2′,3′-Dialdehydo-UDP-*N*-acetylglucosamine inhibits UDP-*N*-acetylglucosamine 2-epimerase, the key enzyme of sialic acid biosynthesis. FEBS Lett..

[130] Al-Rawi S., Hinderlich S., Reutter W., Giannis A. (2004). Synthesis and biochemical properties of reversible inhibitors of UDP-*N*-acetylglucosamine 2-epimerase. Angew. Chem. Int. Ed..

[131] Matsushita T., Sati G.C., Kondasinghe N., Pirrone M.G., Kato T., Waduge P., Kumar H.S., Sanchon A.C., Dobosz-Bartoszek M., Shcherbakov D. (2019). Design, multigram synthesis, and in vitro and in vivo evaluation of propylamycin: A semisynthetic 4,5-deoxystreptamine class aminoglycoside for the treatment of drug-resistant Enterobacteriaceae and other Gram-negative pathogens. J. Am. Chem. Soc..

[132] Duscha S., Boukari H., Shcherbakov D., Salian S., Silva S., Kendall A., Kato T., Akbergenov R., Perez-Fernandez D., Bernet B. (2014). Identification and evaluation of improved 4′-*O*-(alkyl) 4,5-disubstituted 2-deoxystreptamines as next-generation aminoglycoside antibiotics. mBio.

[133] Büll C., Boltje T.J., van Dinther E.A.W., Peters T., de Graaf A.M.A., Leusen J.H.W., Kreutz M., Figdor C.G., den Brok M.H., Adema G.J. (2015). Targeted delivery of a sialic acid-blocking glycomimetic to cancer cells inhibits metastatic spread. ACS Nano.

[134] Büll C., Boltje T.J., Wassink M., de Graaf A.M.A., van Delft F.L., den Brok M.H., Adema G.J. (2016). Targeting aberrant sialylation in cancer cells using a fluorinated sialic acid analog impairs adhesion, migration, and in vivo tumor growth. Mol. Cancer Ther..

[135] Zhu J.-S., McCormick N.E., Timmons S.C., Jakeman D.L. (2016). Synthesis of α-deoxymono and difluorohexopyranosyl 1-phosphates and kinetic evaluation with thymidylyl- and guanidylyltransferases. J. Org. Chem..

[136] Hanzawa Y., Uda J., Kobayashi Y., Ishido Y., Taguchi T., Shiro M. (1991). Trifluoromethylation of chiral aldehyde and synthesis of 6-deoxy-6,6,6-trifluorohexoses. Chem. Pharm. Bull..

[137] Achmatowicz M.M., Allen J.G., Bio M.M., Bartberger M.D., Borths C.J., Colyer J.T., Crockett R.D., Hwang T.-L., Koek J.N., Osgood S.A. (2016). Telescoped process to manufacture 6,6,6-trifluorofucose via diastereoselective transfer hydrogenation: Scalable access to an inhibitor of fucosylation utilized in monoclonal antibody production. J. Org. Chem..

[138] Allen J.G., Mujacic M., Frohn M.J., Pickrell A.J., Kodama P., Bagal D., San Miguel T., Sickmier E.A., Osgood S., Swietlow A. (2016). Facile modulation of antibody fucosylation with small molecule fucostatin inhibitors and cocrystal structure with GDP-mannose 4,6-dehydratase. ACS Chem. Biol..

[139] Okeley N.M., Alley S.C., Anderson M.E., Boursalian T.E., Burke P.J., Emmerton K.M., Jeffrey S.C., Klussman K., Law C.-L., Sussman D. (2013). Development of orally active inhibitors of protein and cellular fucosylation. Proc. Natl. Acad. Sci. USA.

[140] Li J., Hsu H.-C., Ding Y., Li H., Wu Q., Yang P., Luo B., Rowse A.L., Spalding D.M., Bridges S.L. (2014). Inhibition of fucosylation reshapes inflammatory macrophages and suppresses type II collagen-induced arthritis. Arthritis Rheumatol..

[141] Belcher J.D., Chen C., Nguyen J., Abdulla F., Nguyen P., Nguyen M., Okeley N.M., Benjamin D.R., Senter P.D., Vercellotti G.M. (2015). The fucosylation inhibitor, 2-fluorofucose, inhibits vaso-occlusion, leukocyte-endothelium interactions and NF-κB activation in transgenic sickle mice. PLoS ONE.

[142] Soriano del Amo D., Wang W., Besanceney C., Zheng T., He Y., Gerwe B., Seidel R.D., Wu P. (2010). Chemoenzymatic synthesis of the sialyl Lewis X glycan and its derivatives. Carbohydr. Res..

[143] Wang W., Hu T., Frantom P.A., Zheng T., Gerwe B., Soriano del Amo D., Garret S., Seidel R.D., Wu P. (2009). Chemoenzymatic synthesis of GDP-L-fucose and the Lewis X glycan derivatives. Proc. Natl. Acad. Sci. USA.

[144] Ichikawa Y., Lin Y.-C., Dumas D.P., Shen G.-J., Garcia-Junceda E., Williams M.A., Bayer R., Ketcham C., Walker L.E., Paulson J.C. (1992). Chemical-enzymatic synthesis and conformational analysis of sialyl Lewis x and derivatives. J. Am. Chem. Soc..

[145] Lee H.-Y., Chen C.-Y., Tsai T.-I., Li S.-T., Lin K.-H., Cheng Y.-Y., Ren C.-T., Cheng T.-J.R., Wu C.-Y., Wong C.-H. (2014). Immunogenicity study of Globo H analogues with modification at the reducing or nonreducing end of the tumor antigen. J. Am. Chem. Soc..

[146] Zhang Y., Liu Y., Wang Z., Wang Z., Huang L. (2012). The synthesis of a 2-deoxy-2-acetonyl sugar from its corresponding natural saccharide. J. Chem. Res..

[147] Hang H.C., Bertozzi C.R. (2001). Ketone isosteres of 2-*N*-acetamidosugars as substrates for metabolic cell surface engineering. J. Am. Chem. Soc..

[148] Cai L., Guan W., Chen W., Wang P.G. (2010). Chemoenzymatic synthesis of uridine 5′-diphospho-2-acetonyl-2-deoxy-α-D-glucose as C2-carbon isostere of UDP-GlcNAc. J. Org. Chem..

[149] Rawat M., Newton G.L., Ko M., Martinez G.J., Fahey R.C., Av-Gay Y. (2002). Mycothiol-deficient Mycobacterium smegmatis mutants are hypersensitive to alkylating agents, free radicals, and antibiotics. Antimicrob. Agents Chemother..

[150] Gammon D.W., Hunter R., Steenkamp D.J., Mudzunga T.T. (2003). Synthesis of 2-deoxy-2-C-alkylglucosides of *myo*-inositol as possible inhibitors of a *N*-deacetylase enzyme in the biosynthesis of mycothiol. Bioorg. Med. Chem..

[151] Abdelwahab N.Z., Crossman A.T., Sullivan L., Ferguson M.A.J., Urbaniak M.D. (2012). Inhibitors incorporating zinc-binding groups target the GlcNAc-PI de-*N*-acetylase in *Trypanosoma brucei*, the causative agent of African sleeping sickness. Chem. Biol. Drug Des..

[152] Mehlert A., Zitzmann N., Richardson J.M., Treumann A., Ferguson M.A.J. (1998). The glycosylation of the variant surface glycoproteins and procyclic acidic repetitive proteins of *Trypanosoma brucei*. Mol. Biochem. Parasitol..

[153] Urbaniak M.D., Crossman A., Chang T., Smith T.K., van Aalten D.M.F., Ferguson M.A.J. (2005). The *N*-acetyl-D-glucosaminylphosphatidylinositol de-*N*-acetylase of glycosylphosphatidylinositol biosynthesis is a zinc metalloenzyme. J. Biol. Chem..

[154] Abdelwahab N.Z., Urbaniak M.D., Ferguson M.A.J., Crossman A.T. (2011). Synthesis of potential metal-binding group compounds to examine the zinc dependency of the GPI de-*N*-acetylase metalloenzyme in *Trypanosoma brucei*. Carbohydr. Res..

[155] DiFrancesco B.R., Morrison Z.A., Nitz M. (2018). Monosaccharide inhibitors targeting carbohydrate esterase family 4 de-*N*-acetylases. Bioorg. Med. Chem..

[156] Crous W., Naidoo K.J. (2016). Conformational and electrostatic analysis of S_N_1 donor analogue glycomimetic inhibitors of ST3Gal-I mammalian sialyltransferase. Bioorg. Med. Chem..

[157] Izumi M., Wada K., Yuasa H., Hashimoto H. (2005). Synthesis of bisubstrate and donor analogues of sialyltransferase and their inhibitory activities. J. Org. Chem..

[158] Büll C., Heise T., Adema G.J., Boltje T.J. (2016). Sialic acid mimetics to target the sialic acid-Siglec axis. Trends Biochem. Sci..

[159] Bradley S.J., Fazli A., Kiefel M.J., von Itzstein M. (2001). Synthesis of novel sialylmimetics as biological probes. Bioorg. Med. Chem. Lett..

[160] Fazli A., Bradley S.J., Kiefel M.J., Jolly C., Holmes I.H., von Itzstein M. (2001). Synthesis and biological evaluation of sialylmimetics as rotavirus inhibitors. J. Med. Chem..

[161] Hoos R., Huixin J., Vasella A., Weiss P. (1996). Synthesis and enzymatic evaluation of substrates and inhibitors of β-glucuronidases. Helv. Chim. Acta.

[162] Mochizuki T., Iwano Y., Shiozaki M., Kurakata S., Kanai S., Nishijima M. (2000). Lipid A-type pyrancarboxylic acid derivatives, their synthesis and their biological activities. Tetrahedron.

[163] Downey A.M., Cairo C.W. (2013). α-Bromophosphonate analogs of glucose-6-phosphate are inhibitors of glucose-6-phosphatase. Carbohydr. Res..

[164] Kim-Muller J.Y., Accili D. (2011). Selective insulin sensitizers. Science.

[165] Ashmore J., Hastings A.B., Nesbett F.B. (1954). The effect of diabetes and fasting on liver glucose-6-phosphatase. Proc. Natl. Acad. Sci. USA.

